# SARS‐CoV‐2‐infected human airway epithelial cell cultures uniquely lack interferon and immediate early gene responses caused by other coronaviruses

**DOI:** 10.1002/cti2.1503

**Published:** 2024-04-15

**Authors:** Ying Wang, Melissa Thaler, Clarisse Salgado‐Benvindo, Nathan Ly, Anouk A Leijs, Dennis K Ninaber, Philip M Hansbro, Fia Boedijono, Martijn J van Hemert, Pieter S Hiemstra, Anne M van der Does, Alen Faiz

**Affiliations:** ^1^ PulmoScience Lab, Department of Pulmonology Leiden University Medical Center Leiden The Netherlands; ^2^ Department of Medical Microbiology Leiden University Medical Center Leiden The Netherlands; ^3^ Respiratory Bioinformatics and Molecular Biology (RBMB), School of Life Sciences University of Technology Sydney Sydney NSW Australia; ^4^ Centre for Inflammation Centenary Institute and University of Technology Sydney, Faculty of Science Sydney NSW Australia

**Keywords:** coronavirus, immediate early genes, primary airway epithelial cells, RNA sequencing, SARS‐CoV‐2

## Abstract

**Objectives:**

Severe acute respiratory syndrome coronavirus 2 (SARS‐CoV‐2) is a member of a class of highly pathogenic coronaviruses. The large family of coronaviruses, however, also includes members that cause only mild symptoms, like human coronavirus‐229E (HCoV‐229E) or OC43 (HCoV‐OC43). Unravelling how molecular (and cellular) pathophysiology differs between highly and low pathogenic coronaviruses is important for the development of therapeutic strategies.

**Methods:**

Here, we analysed the transcriptome of primary human bronchial epithelial cells (PBEC), differentiated at the air–liquid interface (ALI) after infection with SARS‐CoV‐2, SARS‐CoV, Middle East Respiratory Syndrome (MERS)‐CoV and HCoV‐229E using bulk RNA sequencing.

**Results:**

ALI‐PBEC were efficiently infected by all viruses, and SARS‐CoV, MERS‐CoV and HCoV‐229E infection resulted in a largely similar transcriptional response. The response to SARS‐CoV‐2 infection differed markedly as it uniquely lacked the increase in expression of immediate early genes, including *FOS*, *FOSB* and *NR4A1* that was observed with all other coronaviruses. This finding was further confirmed in publicly available experimental and clinical datasets. Interfering with NR4A1 signalling in Calu‐3 lung epithelial cells resulted in a 100‐fold reduction in extracellular RNA copies of SARS‐CoV‐2 and MERS‐CoV, suggesting an involvement in virus replication. Furthermore, a lack in induction of interferon‐related gene expression characterised the main difference between the highly pathogenic coronaviruses and low pathogenic viruses HCoV‐229E and HCoV‐OC43.

**Conclusion:**

Our results demonstrate a previously unknown suppression of a host response gene set by SARS‐CoV‐2 and confirm a difference in interferon‐related gene expression between highly pathogenic and low pathogenic coronaviruses.

## Graphical abstract

In this study, we found that SARS‐CoV‐2, in contrast to other coronaviruses, suppressed the induction of an immediate early gene set by human well‐differentiated bronchial epithelial cultures. Furthermore, a significant difference in interferon‐related gene expression was detected between highly pathogenic and low pathogenic coronaviruses upon infection of these cultures.

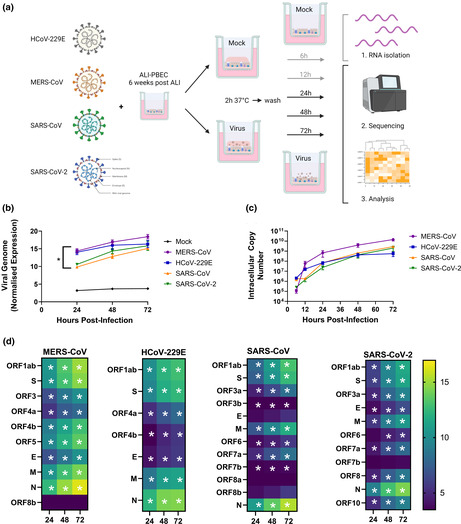

## Introduction

The outbreak of severe acute respiratory syndrome coronavirus 2 (SARS‐CoV‐2) that started in late 2019 constituted an enormous threat to human health worldwide. Together with other highly pathogenic coronaviruses such as SARS‐CoV and Middle East respiratory syndrome coronavirus (MERS‐CoV), SARS‐CoV‐2 belongs to the genus *Betacoronavirus* (β‐CoV), of the Coronaviridae family. These coronaviruses share many similarities but also exhibit evident differences. For human coronaviruses, the primary site of infection is the respiratory tract. However, there are marked differences in their host entry requirements, pathogenicity and transmissibility.^1^ For example, the use of different host receptors for viral entry helps to explain differences in tissue tropism. SARS‐CoV‐2 furthermore demonstrates higher transmissibility, a wider range of clinical symptoms and lower mortality rates than SARS‐CoV and MERS‐CoV.^1^, ^2^ Not all human coronaviruses cause severe clinical symptoms. In fact, many of them only cause mild symptoms in healthy individuals. Human coronavirus 229E (HCoV‐229E),^3^ a member of the *Alphacoronavirus* (α‐CoV) genus, or human coronavirus OC43 (HCoV‐OC43),^4^ a member of the *Betacoronavirus* genus, are two of the causative agents of the common cold. While HCoV‐229E and HCoV‐OC43 cause only mild symptoms in the upper respiratory tract of healthy people, SARS‐CoV, SARS‐CoV‐2 and MERS‐CoV may also infect the lower respiratory tract, resulting in a much wider range of respiratory illnesses and other non‐pulmonary clinical manifestations that can be life threatening. It is important for the development of therapeutic strategies against infection with SARS‐CoV‐2, but also other (future) pathogenic coronaviruses, to understand the differences in their unique pathological characteristics.

RNA sequencing studies have shed light on many aspects of SARS‐CoV‐2 pathobiology. For example, bulk and single‐cell RNA sequencing revealed the spatial distribution of cell entry factor expression and cell tropism (infection biology) within the respiratory epithelium.^5^, ^6^, ^7^, ^8^ Transcriptional analysis also showed differences between mild and severe COVID‐19 cases and the diversity in immune responses.^9^, ^10^ Furthermore, infection of ciliated cells, the main cell type targeted by SARS‐CoV‐2, was demonstrated to lead to a significant downregulation of cilium assembly and motility pathways.^8^

Despite our increasing knowledge on SARS‐CoV‐2 infection biology, there are still important gaps in our understanding of what distinguishes SARS‐CoV‐2 from other coronaviruses in terms of pathogenesis and transmission. Comparing host responses during the initial phase of the infection of the respiratory epithelium between SARS‐CoV‐2 and other (highly pathogenic and low pathogenic) coronaviruses may provide important clues on how SARS‐CoV‐2 establishes its distinct effects.

Recent research has compared infectivity of various coronavirus strains in human nasal epithelial cultures,^11^ but this study did not provide insight into host transcriptional responses and was limited to the nasal region. Others have compared transcriptional responses to coronavirus infection by combining several independent datasets,^12^ or with only a limited number of different coronaviruses.^13^ Here, we sought to determine the differences in the transcriptional response of well‐differentiated primary bronchial epithelial cell cultures to infection with highly pathogenic SARS‐CoV, SARS‐CoV‐2, MERS‐CoV and low pathogenic HCoV‐229E and in specific experiments also HCoV‐OC43. Paired comparative analysis revealed differences between the host responses to these viruses and uncovered modulations of signalling pathways that were unique to SARS‐CoV‐2 infection.

## Results

### Successful infections of primary airway epithelial cells with SARS‐CoV, SARS‐CoV‐2, MERS‐CoV or HCoV‐229E

**Figure 1:**
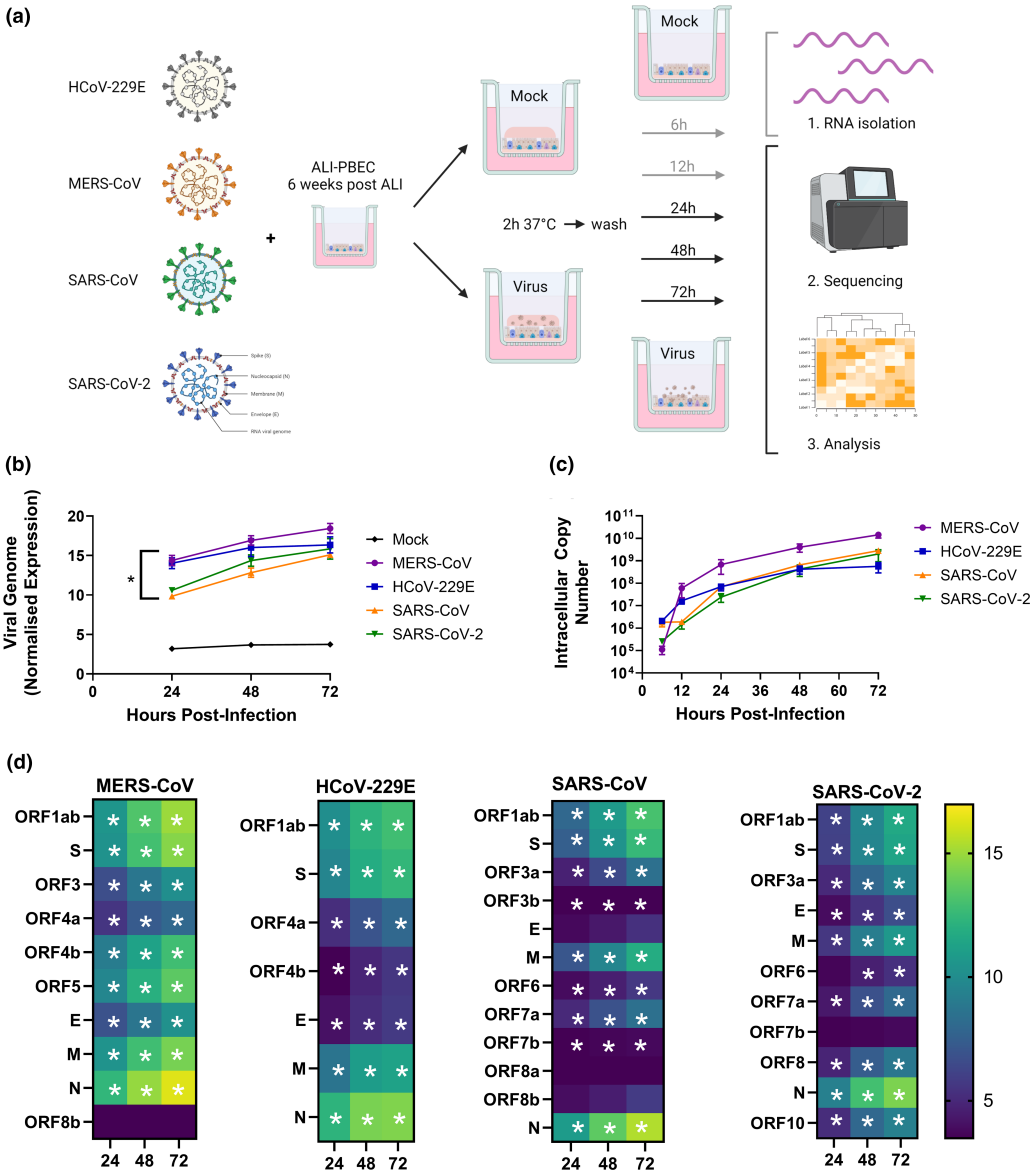
Experimental design and replication kinetics of several coronaviruses in ALI‐PBEC. **(a)** Schematic diagram of experimental design and infection protocol. ALI‐PBEC that were differentiated for 6 weeks were infected in parallel with four different coronaviruses. At several time points after infection, RNA was harvested from these cultures and RNA from the 24, 48 and 72 h time points was analysed by bulk RNA‐seq. **(b)** Replication of coronaviruses in ALI‐PBEC over the 72 h period was assessed by mapping the viral sequences in the bulk RNA‐seq dataset. Significant differences between virus and mock for all time points were assessed using a two‐way ANOVA followed by an unprotected Fisher's least significance difference test, and **(c)** intracellular viral RNA copies at each time point were measured by RT‐qPCR. **(d)** Viral reads were mapped against the respective genome sequence and changes in the abundance of sequences mapping to the various viral open reading frames during infection are summarised. Statistics were conducted using a paired edgeR differential expression analysis comparing all viruses to mock. Data are shown as mean ± SEM of cultures derived from *n* = 4 different donors, and differences were considered significant at *P < 0.05. Panel **a** was created with BioRender.com.

To compare the transcriptional response of airway epithelial cells to various coronaviruses, we infected 6‐week‐differentiated ALI‐PBEC^14^ from four donors with an equal amount (~30 000 PFU) of either highly pathogenic SARS‐CoV, SARS‐CoV‐2 or MERS‐CoV, or low pathogenic HCoV‐H229E. The relatively low amount of PFU was chosen to be representative of an initial infection (and subsequent spread over the tissue). Cultures were harvested at 6, 12, 24, 48 and 72 hpi followed by RNA isolation. RNA collected at 24, 48 and 72 hpi was subsequently used to perform bulk RNA‐seq analysis (workflow is depicted in Figure 1a). Viral replication was assessed by analysis of the RNA‐seq datasets at 24, 48 and 72 hpi and additionally for each harvested time point (6, 12, 24, 48 and 72 hpi) by quantitative real‐time PCR (RT‐qPCR) analysis. The normalised viral genome counts (measured by RNA‐seq) of all coronaviruses showed a significant increase over the 72 h incubation period, with MERS‐CoV having the highest observed increase (Figure 1b). These results were confirmed by quantification of intracellular viral RNA copies by RT‐qPCR (Figure 1c). Furthermore, expression of genes encoding structural and non‐structural proteins were increasing during infection time (Figure 1d) confirming active replication. A strong increase in subgenomic RNA encoding the most abundant viral protein N was observed for infections with all coronaviruses (Figure 1d).

### Gene expression of entry‐related factors is changed during coronavirus infection of airway epithelial cells

**Figure 2:**
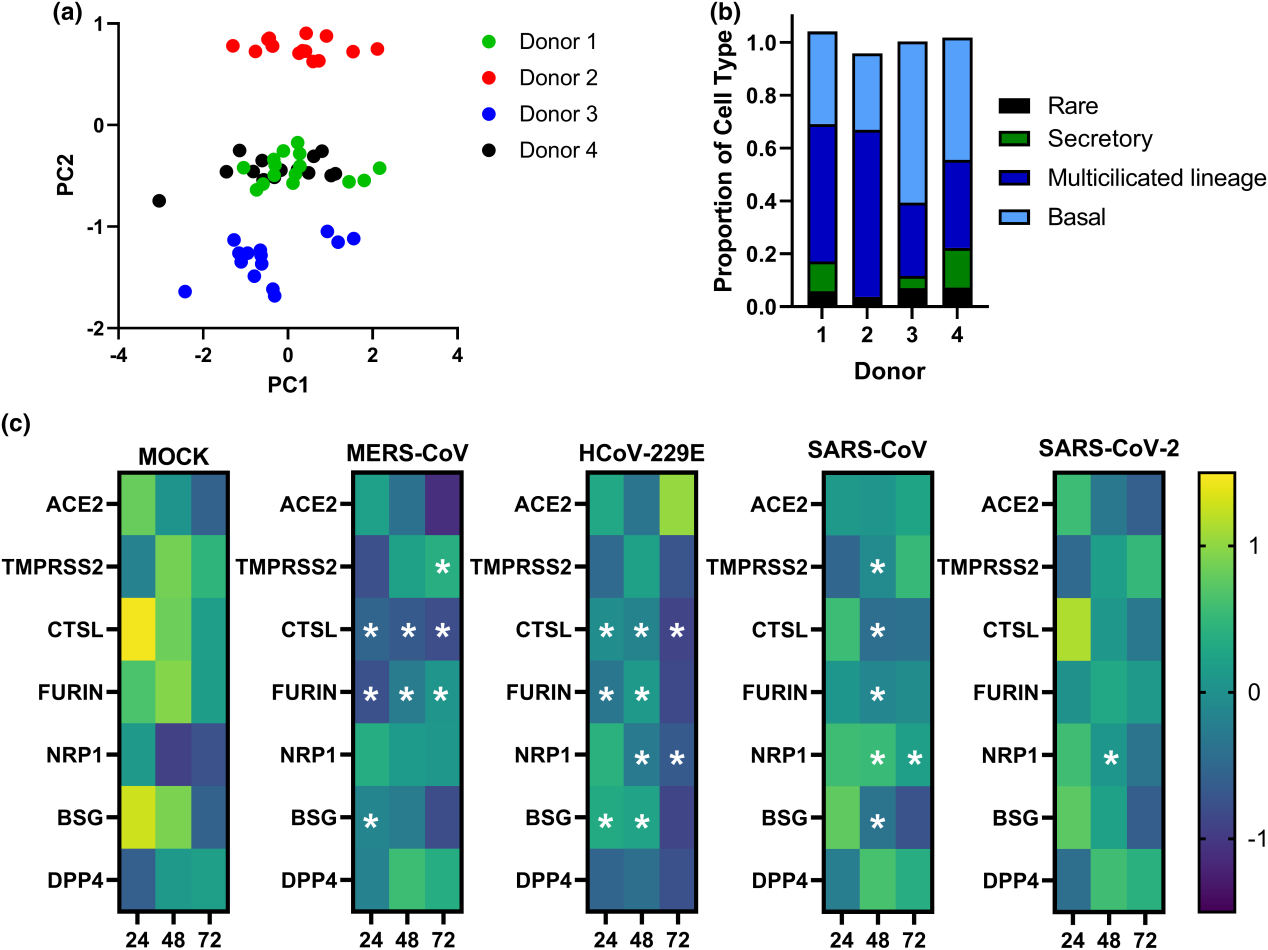
Donor‐dependent clustering of samples, cellular composition and expression of viral entry‐related genes in well‐differentiated ALI‐PBEC. PBEC that were differentiated for 6 weeks at ALI were infected in parallel with four different coronaviruses. **(a)** PCA of transcriptional profiles of all cell culture samples over time of infection. **(b)** Relative proportion of different cell types for each donor determined by cellular deconvolution of the transcriptomic dataset. **(c)** Changes in expression of common viral entry‐related genes over time of infection (*n* = 4). Statistics were conducted using a paired edgeR differential expression analysis comparing all viruses to mock where a *P‐value < 0.05 was considered significant.

A principal component analysis (PCA) of the complete RNA‐seq dataset demonstrated that samples clustered based on donor ID (Figure 2a). This finding is not unexpected and could be explained by differences in cell composition of the cultured airway epithelium between donors. Cellular deconvolution of these samples showed that each donor had a distinct profile of ciliated, mucosecretory and basal cells (Figure 2b), which was not altered by the different coronavirus infections (Supplementary figure 1). Next, we focused on the expression of known coronavirus entry‐related factors and determined whether changes occurred during infection (Figure 2c). Overall, we mostly observed a decrease in expression of these genes during infection, except for SARS‐CoV‐2. Angiotensin‐converting enzyme 2 (*ACE2*) and transmembrane serine protease 2 (*TMPRSS2*), the genes encoding for the main viral entry receptor and protease that cleaves the SARS‐CoV‐2 S‐protein for subsequent cell entry, respectively, were not significantly altered in most of our samples. Significant changes were only found for *TMPRSS2* at later time points in SARS‐CoV (48 hpi) and MERS‐CoV (72 hpi) infection compared to mock‐infected controls. Cathepsin L (*CTSL*) expression declined significantly over time in MERS‐CoV and HCoV‐229E‐infected cultures, while *Furin* expression increased. Neuropilin‐1 (*NRP1*), which was described as a co‐receptor for SARS‐CoV‐2 cell entry^15^, showed an increase in expression at 24 and 48 h after SARS‐CoV‐2 infection but then declined at 72 hpi (Figure 2c). Together these data indicate that infection results in shifts in expression of various factors involved in coronavirus entry, which may mediate further spread upon initial infection in the epithelial cultures.

### Increased immediate early gene expression by airway epithelial cells infected with SARS‐CoV, MERS‐CoV and HCoV‐229E

**Figure 3:**
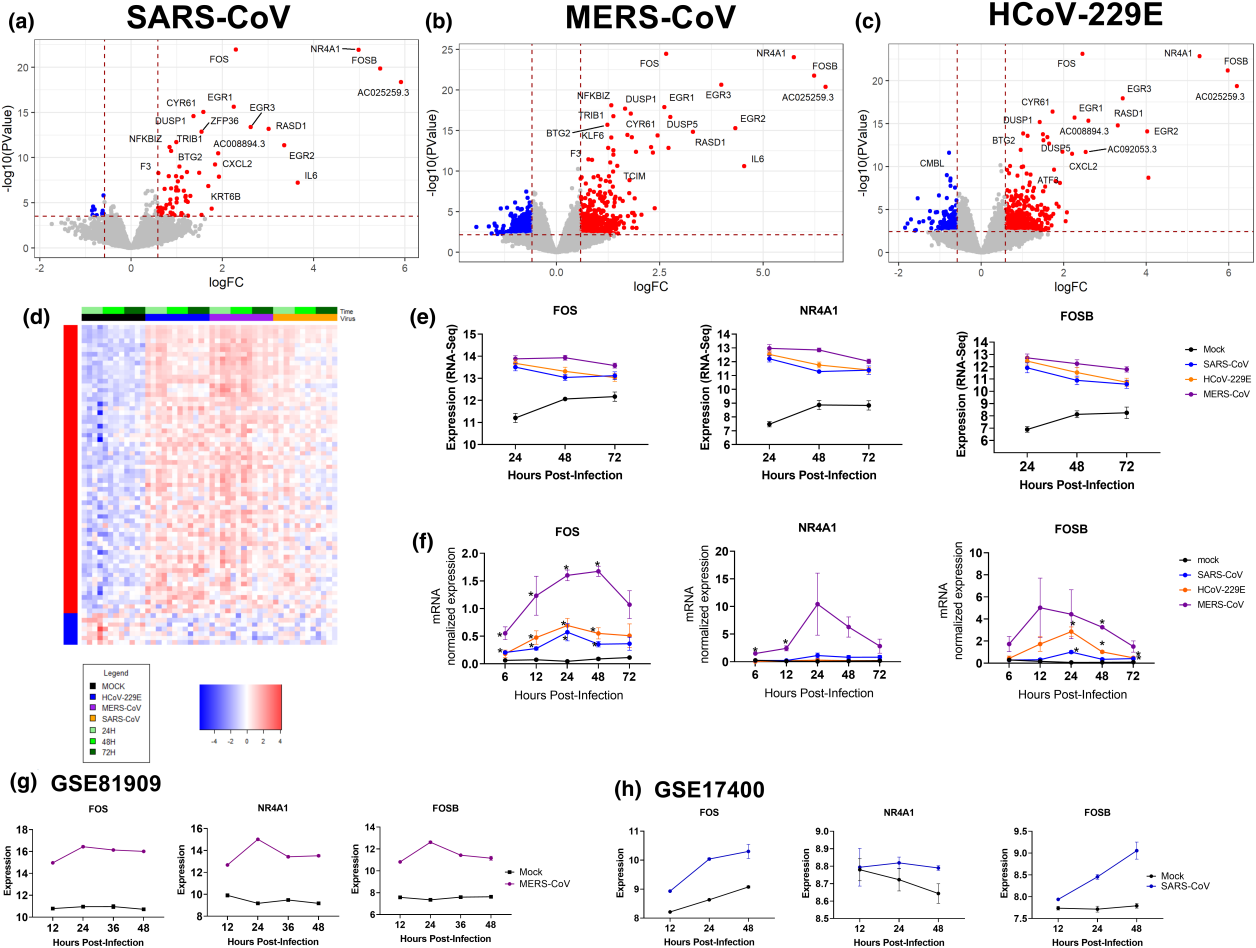
Gene expression profiles of well‐differentiated primary bronchial epithelial cell cultures infected with three different coronaviruses. **(a)** Volcano plots depicting gene expression profiles of bronchial epithelial cell cultures infected with **(a)** SARS‐CoV, **(b)** MERS‐CoV or **(c)** HCoV‐229E at 24 hpi in comparison to the uninfected controls. Red dots indicate significantly upregulated genes, and blue dots indicate significantly downregulated genes. **(d)** Heatmap of significantly changed genes during infection with SARS‐CoV, MERS‐CoV or HCoV‐229E and mock as uninfected control (log_2_fold change > |1.5| and FDR < 0.05). **(e)** Changes in gene expression of FOS, NR4A1 and FOSB during infection with three different coronaviruses using data from the RNA‐seq analysis. **(f)** The same genes as in figure **e** were analysed for all harvested time points using traditional qPCR. Data are expressed as normalised values for *ATP5B* and *OAZ1*. **(g)** A publicly available microarray dataset (GSE81909) was used to analyse the impact of MERS‐CoV infection on these three IEGs in primary airway epithelial cells. **(h)** A publicly available dataset (GSE17400) was assessed to analyse the effect of SARS‐CoV infection on these IEGs in Calu‐3 cells during 48 h with an MOI of 0.1. Data in **a–f** are from *n* = 4 different donors. Significant differences between virus infection and mock for all time points were assessed using a two‐way ANOVA followed by an unprotected Fisher's least significance difference test, *P < 0.05 was considered significant, data in **e–h** are depicted as mean ± SEM.

Before addressing the SARS‐CoV‐2 transcriptional profiles, we first compared the expression profiles of ALI‐PBEC in response to SARS‐CoV, MERS‐CoV and HCoV‐229E at 24, 48 and 72 hpi. ALI‐PBEC infected with SARS‐CoV showed significant different expressions of 80 genes at 24 hpi, compared to the control, of which 69 were increased in expression and 11 decreased in expression. In MERS‐CoV‐infected cells, we identified 934 differentially expressed genes, of which 580 were increased and 354 decreased in expression at 24 hpi. Cells infected with HCoV‐229E displayed an increased expression of 433 genes and reduced expression of 162 genes at 24 hpi (FDR < 0.05, FC > 1.5). The significantly changed genes at 24 hpi are depicted in volcano plots in Figure 3a–c and Supplementary table 3 (results of the other time points – 48 and 72 h – are included in Supplementary figure 2) and the total expression profiles over time in Figure 3d. A specific set of genes (see the Methods section) was significantly increased at all timepoints after start of infection with SARS‐CoV, MERS‐CoV and HCoV‐229E. Many of these belong to a group of well‐known genes, called immediate early genes (IEGs), which play an important role in the cell's rapid response to its external environment.^16^ The remarkable overlap in these genes between ALI‐PBEC infected with SARS‐CoV, MERS‐CoV or HCoV‐229E (Figure 3e, Supplementary figure 2) was dominated by *FOS*, *NR4A1* and *FOSB* gene expression, and their increased expression was extracted from the RNA‐seq dataset (Figure 3e) and supported by RT‐qPCR analysis of these genes (Figure 3f). To confirm our findings, we investigated *FOS*, *NR4A1* and *FOSB* gene expression also in two publicly available datasets of primary airway epithelial cells and Calu‐3 cells infected with MERS‐CoV and SARS‐CoV, respectively (Figure 3g and h). These datasets supported our results showing upregulation of *FOS*, *NR4A1* and *FOSB* gene expression following MERS‐CoV and SARS‐CoV infection (Figure 3g and h). Together these results indicate an activation of the expression of specific IEGS by these coronaviruses and their sustained high level of expression for at least 72 hpi.

### Airway epithelial cells infected with SARS‐CoV‐2 display a distinct transcriptional response that lacks induction of immediate early genes

**Figure 4:**
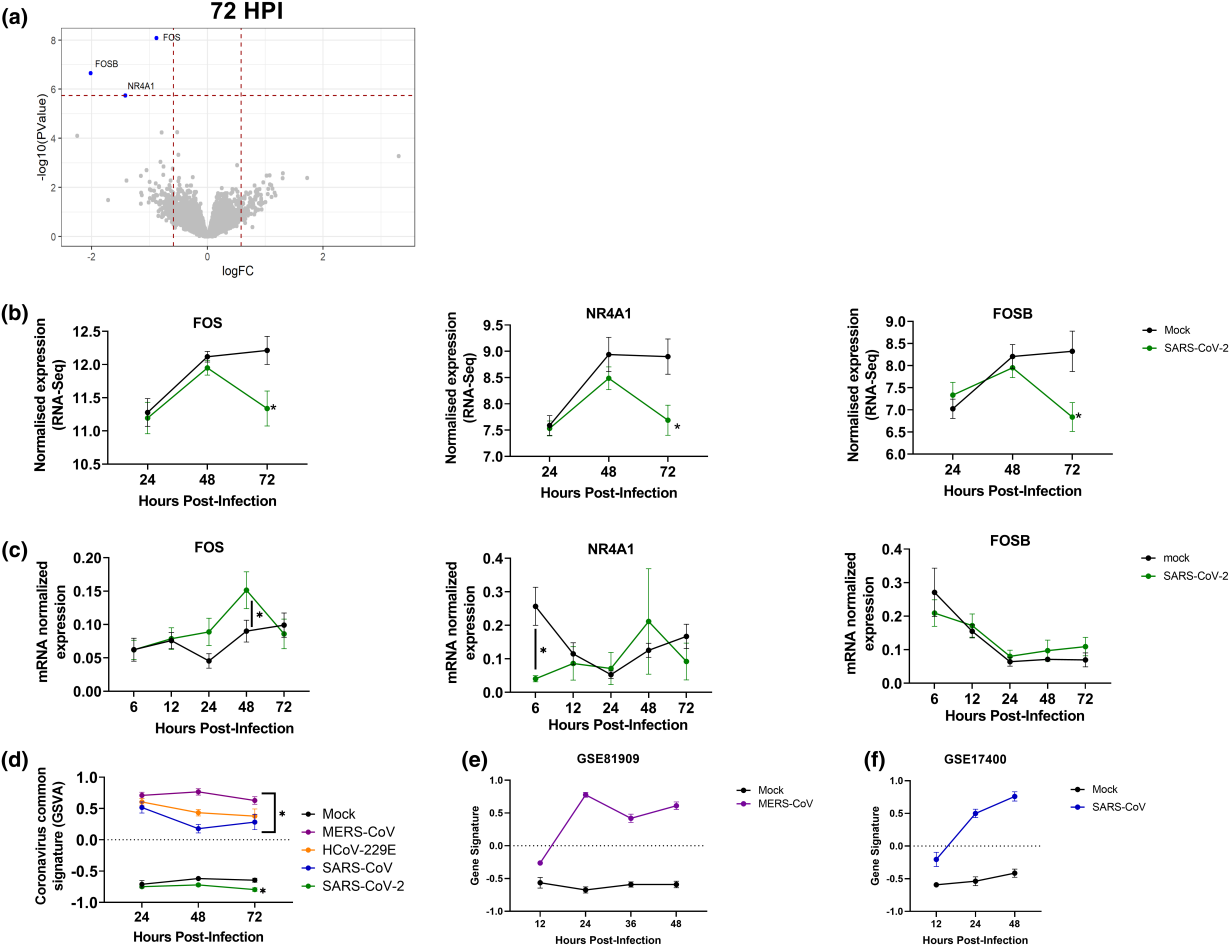
Gene expression profiles of well‐differentiated primary bronchial epithelial cells during SARS‐CoV‐2 infection. **(a)** Volcano plot depicting gene expression profiles of bronchial epithelial cell cultures infected with SARS‐CoV‐2 at 72 hpi (*n* = 4). **(b)** The expression of FOS, *NR4A1* and *FOSB* over a 72 h infection period with SARS‐CoV‐2 using data from the RNA‐seq analysis (*n* = 4). **(c)** The same genes as in figure **b** were analysed for all time points using traditional qPCR. **(d)** Comparison of an MERS‐CoV, SARS‐CoV, HCoV‐229E signature gene set between all coronavirus‐infected cultures at 24, 48 and 72 hpi (*n* = 4). **(e)** A publicly available microarray dataset (GSE81909) was assessed to analyse the impact of MERS‐CoV infection on the MERS‐CoV, SARS‐CoV, HCoV‐229E signature gene set. **(f)** A publicly available dataset (GSE17400) was assessed to analyse the effect of SARS‐CoV infection on the MERS‐CoV, SARS‐CoV, HCoV‐229E signature gene set. Significant differences between virus infection and mock for all time points were assessed using a two‐way ANOVA followed by an unprotected Fisher's least significance difference test, *P < 0.05 was considered significant, data in **b–f** are depicted as mean ± SEM.

Next, we addressed the specific transcriptional responses of ALI‐PBEC cultures following SARS‐CoV‐2 infection. Strikingly, gene expression analysis identified that despite a viral load that was comparable to the other viruses, no genes were significantly changed at 24 and 48 hpi (data not shown). At 72 hpi, there were no significantly increased genes while a decrease in three genes was observed (*FOS*, *NR4A1* and *FOSB*) (Figure 4a, Supplementary table 4). These results show that SARS‐CoV‐2‐infected ALI‐PBEC lacks the increased IEG expression that was observed for the other coronaviruses, while even a decrease in the gene expression of *FOS*, *NR4A1* and *FOSB* in SARS‐CoV‐2‐infected ALI‐PBEC was observed (Figure 4b). Using RT‐qPCR, we confirmed that over the time course of 6–72 hpi there was no increase in expression of these genes (Figure 4c). Only *FOS* mRNA expression was increased at 48 hpi compared to mock infection. However, compared to the other three coronaviruses, the upregulation of *FOS* expression by SARS‐CoV‐2 was minimal, and not supported by the results of the RNA‐seq data. Furthermore, we conducted an analysis of signature genes increased by SARS‐CoV, MERS‐CoV and HCoV‐229E at all timepoints (included genes are listed in the Methods section). It was evident from this analysis that SARS‐CoV‐2 did not increase these genes to the same extent as the other coronaviruses did and even appeared to actively suppress them compared to the control group (Figure 4d). This signature gene set was furthermore validated to be changed in MERS‐CoV and SARS‐CoV infections reported in publicly available datasets (Figure 4e and f).

### Validation of the effects of SARS‐CoV‐2 on immediate early gene expression by lung epithelial cells

**Figure 5:**
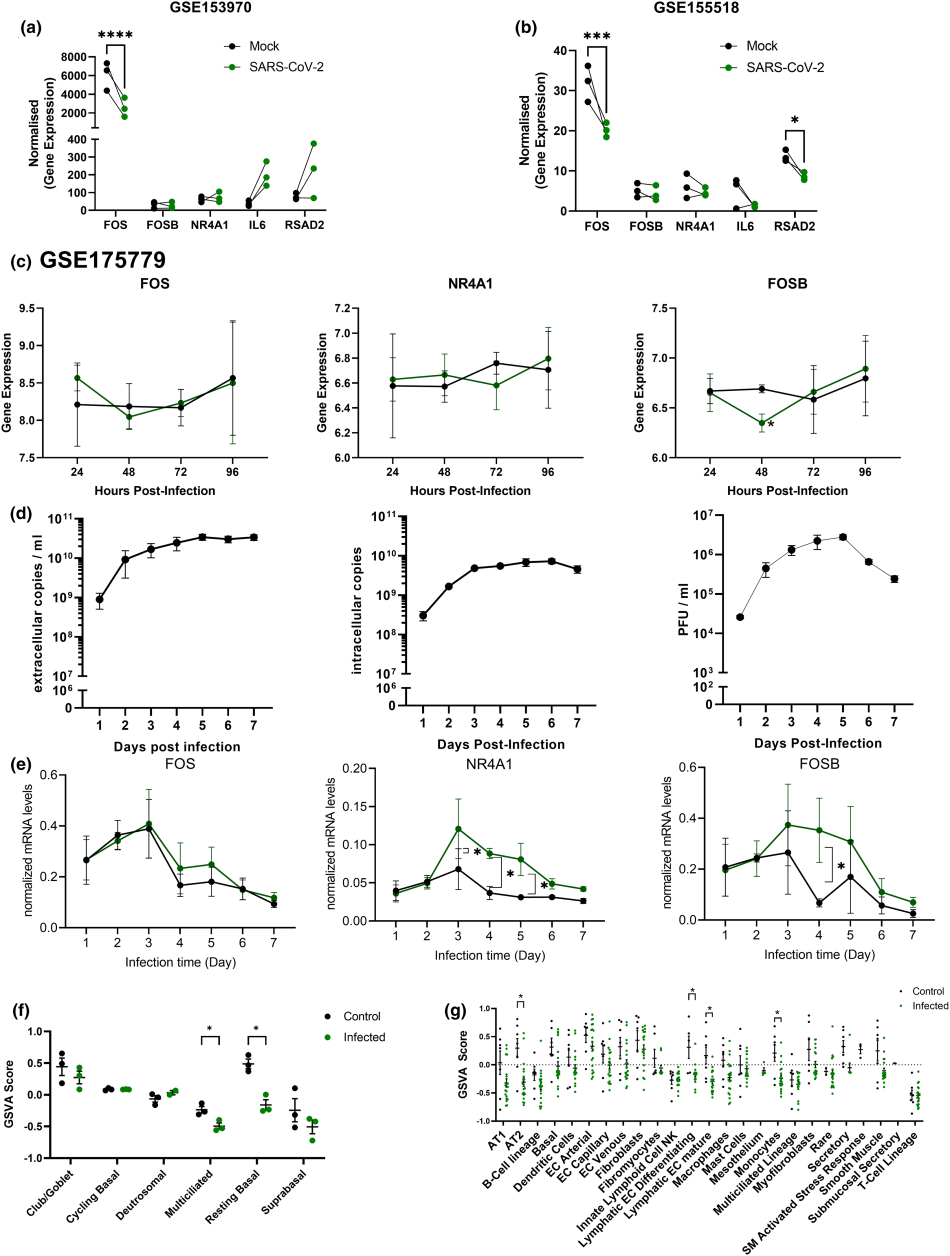
Analysis of IEG expression upon SARS‐CoV‐2 infection of epithelial cells in public datasets and in long‐term infection. **(a)** A publicly available dataset (GSE153970) with RNA‐seq analysis on primary epithelial cell cultures mock‐infected or infected with SARS‐CoV‐2 at an MOI = 0.25 at 48 hpi was analysed for *FOS, NR4A1* and *FOSB* expression. **(b)** A publicly available dataset (GSE155518) derived from primary human lung alveolar epithelial organoid cultures infected with SARS‐CoV‐2 was analysed for *FOS, NR4A1* and *FOSB* expression. **(c)** A publicly available dataset (GSE175779) from primary human bronchial epithelial cells infected with SARS‐CoV‐2 over a 96 h infection period was analysed for *FOS, NR4A1* and *FOSB* expression. **(d)** RT‐qPCR measurement of extracellular (left graph) and intracellular viral RNA (middle graph), and plaque assay measurement of infectious virus particles (right graph) in the apical wash of SARS‐CoV‐2 infected ALI‐PBEC over 7 days; *n* = 3 independent donors. **(e)** The expression of IEGs was measured by qPCR in ALI‐PBEC infected with SARS‐CoV‐2 for 7 days. Data are shown as mean ± SEM; *n* = 3 independent donors. Significant differences between virus infection and mock for all time points were assessed using a two‐way ANOVA followed by an unprotected Fisher's least significance difference test, *P < 0.05 was considered significant, data are depicted as mean ± SEM. **(f)** GSVA analysis of the IEG gene set on a publicly available single‐cell RNA‐Seq dataset^7^ from well‐differentiated human bronchial epithelial cell cultures infected with SARS‐CoV‐2 and analysed 7 days post infection. Significant differences between infected and uninfected cultures were tested using a two‐way ANOVA with Bonferroni correction*P < 0.05 was considered significant, **(g)** GSVA analysis of the IEG gene set on a publicly available Nucleo‐Seq dataset^51^ from cells derived from tissue from deceased COVID‐19 patients and deceased uninfected individuals. Significant differences between infected and uninfected individuals per cell type were tested using an unpaired *t‐*test with Bonferroni correction. *P < 0.05 was considered significant. ***P < 0.001 and ****P < 0.0001.

To better understand and confirm our findings, we further investigated publicly available datasets. First, we started by analysing the expression of the total IEG gene set in three publicly available RNA‐seq datasets, derived from bronchial or alveolar epithelial cells infected with SARS‐CoV‐2 and analysed at various time points post infection, of which none showed a significant change in gene expression compared to the uninfected control (Supplementary figure 3). Next, we analysed these datasets to specifically assess expression of the most prominently changed genes in our own dataset, including *FOS*, *FOSB* and *NR4A1*. In the first dataset, GSE153970, collected from primary human airway epithelial cells cultured at ALI, infected with SARS‐CoV‐2 at an MOI of 0.25 and analysed at 48 hpi, a significant decrease in *FOS* gene expression was found, accompanied by a lack in upregulation of *FOSB* and *NR4A1* (Figure 5a), mirroring our results. In the second dataset, GSE155518, similar results for *FOS* expression were found at 48 hpi in primary lung alveolar type‐2 epithelial cells in a 3D organoid culture infected with SARS‐CoV‐2 (Figure 5b). Finally, we validated our findings using a dataset that originated from a study on SARS‐CoV‐2 infection of ALI‐PBEC that included samples harvested at 6–96 hpi (GSE175779; Figure 5c). This dataset displayed no significant increase in *FOS*, *NR4A1* and *FOSB* gene expression (only a decrease at 48 hpi for *FOSB*) over the 96 h course of infection (Figure 5c), like all other SARS‐CoV‐2 datasets.

To understand whether the observed differences in the changes of these specific genes between SARS‐CoV‐2 and the other coronavirus‐infected cultures were a consequence of a difference in viral load or kinetics, we also analysed cultures that were infected with a higher dose, 10^6^ PFU, of SARS‐CoV‐2 (estimated MOI of 1). We followed gene expression levels in this culture for a longer period, up to 7 days post infection. We assessed viral replication and changes over time using plaque assay and RT‐qPCR. Analysis of infectious virus produced over 7 days showed a peak in the accumulation of infectious progeny at 4–5 days post infection with more than 10^6^ PFU mL^−1^, and a corresponding strong increase in intracellular viral RNA up to 3 days p.i. (Figure 5d). We furthermore detected a significant increase in *NR4A1* expression from 3 to 5 days post infection (Figure 5e) and a slight increase in *FOSB* expression at day 4 after SARS‐CoV‐2 infection, while no effects on *FOS* expression were observed (Figure 5e), suggesting a delayed response to SARS‐CoV‐2. Nevertheless, despite these changes, the increased expression of these genes observed at later time points with a higher MOI was still substantially lower than upon infection with SARS‐CoV, MERS‐CoV or HCoV‐229E in the original experiments where a lower MOI was used. To obtain further insight, we also assessed IEG expression in publicly available datasets of infected cultures and individuals analysed with single‐cell RNA sequencing. In well‐differentiated human PBEC cultures infected with SARS‐CoV‐2 and analysed at 7 days post infection, single‐cell RNA sequencing results showed that resting basal and multi‐ciliated cells had a significantly lower enrichment score for the IEG gene set in infected cells than in non‐infected cells (Figure 5f). Finally, we assessed IEG expression in a clinical dataset that was generated by nucleo‐seq analysis of cells derived from deceased uninfected or SARS‐CoV‐2‐infected individuals. Confirming our findings, no increase in IEG gene expression was observed. In alveolar type‐2 epithelial cells, differentiating and mature lymphatic endothelial cells, and monocytes showed a significantly lower enrichment score in infected than in non‐infected individuals, while secretory epithelial cells showed a strong trend (Figure 5g). Together these results demonstrate that, unlike all other coronaviruses tested, SARS‐CoV‐2 does not induce and might even actively suppress IEG expression in epithelial cells and potentially other cell types.

### NR4A1 antagonist suppresses coronavirus replication

**Figure 6:**
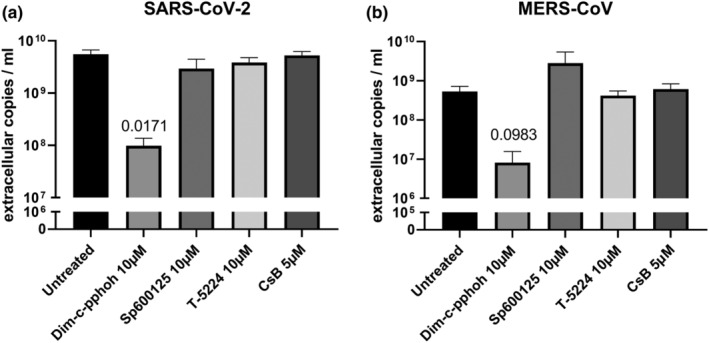
Effect of compounds that modulate pathways related to IEGs on SARS‐CoV‐2 and MERS‐CoV infection. **(a)** Effect of compounds that modulate pathways related to JNK/AP‐1 pathway and NR4A1 on SARS‐CoV‐2 and **(b)** MERS‐CoV infection in Calu‐3 cells. DIM‐C‐pPhOH (NR4A1 antagonist), Sp600125 (JNK inhibitor), T‐5224 (AP‐1 transcription factor inhibitor) and Cytosporone B (CsB; NR4A1 agonist). Calu‐3 cells were preincubated for 1 h with these compounds and subsequently infected with SARS‐CoV‐2 or MERS‐CoV (MOI of 1) in 150 μL of infection medium for 1 h. After 24 h of incubation with compounds, supernatants were harvested. Extracellular copies were measured by RT‐qPCR. Data are shown as mean ± SEM. *n* = 4 independent experiments for SARS‐CoV‐2 and *n* = 3 independent experiments for MERS‐CoV, except for Sp600125 where *n* = 2 independent experiments were performed for both viruses. Statistical significance was tested between a compound and the untreated control using a two‐tailed paired *t*‐test. The *P*‐value is indicated in the graph and is considered significant < 0.05.

Since *FOS*, *FOSB* and *NR4A1* gene expression was consistently lacking in epithelial transcriptional responses to SARS‐CoV‐2, we next investigated their contribution to viral infection by modulation of their activity, or modulation of related signalling pathways, using small molecules. FOS and FOSB are associated with the JNK/AP1 pathway, therefore based on literature, we selected the compounds Sp600125 (JNK inhibitor) and T‐5224 (AP‐1 transcription factor inhibitor) to modulate this pathway. We furthermore selected DIM‐C‐pPhOH (NR4A1 antagonist) and Cytosporone B (NR4A1 agonist) for modulation of the NR4A1 pathway. We compared the effects of these compounds in SARS‐CoV‐2 and MERS‐CoV infected Calu‐3 cells, since the increase in expression of the associated IEG genes was most pronounced after MERS‐CoV infection while it was absent after SARS‐CoV‐2 infection. Sp600125, T‐5224 and Cytosporone B had no effect on viral replication in viral load reduction assays with Calu‐3 lung epithelial cells (Figure 6). Interestingly, DIM‐C‐pPhOH, which inhibits the activity of nuclear receptor 4A1 (NR4A1), resulted in a two‐log reduction of extracellular viral RNA copies for SARS‐CoV‐2 as well as MERS‐CoV infected cells (Figure 6). When we treated uninfected cells in parallel, we observed no cytotoxicity with 10 μm of DIM‐C‐pPhOH (Supplementary figure 4). The inhibitory effect of the modulation of NR4A1 activity on virus replication suggests its possible involvement in infection biology.

### Difference in transcriptional responses between primary bronchial epithelial cultures infected with highly pathogenic and low pathogenic coronaviruses

**Figure 7:**
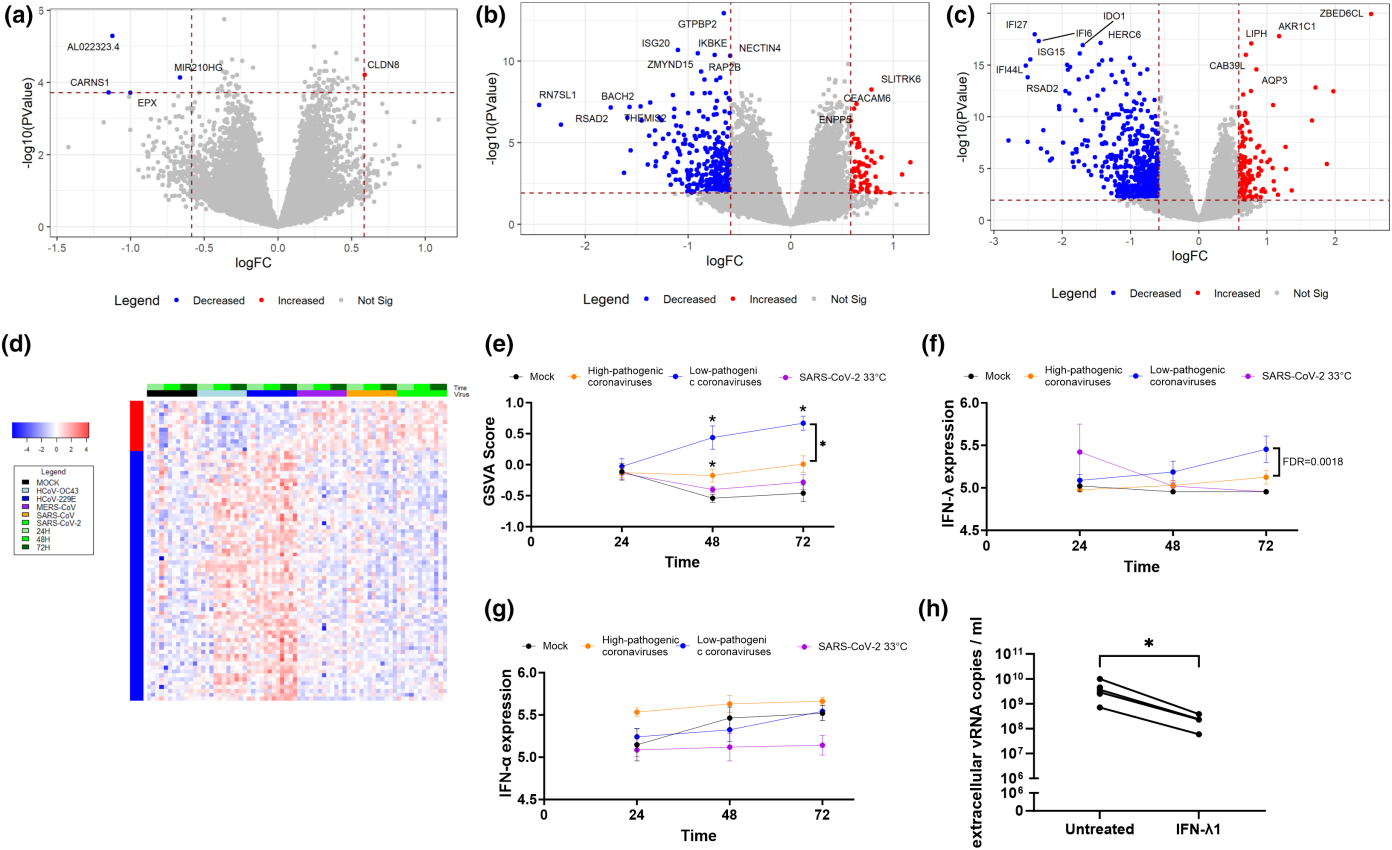
Analysis of interferon (‐response) genes upon coronavirus infection and effects of IFN‐λ1 supplementation on viral load. **(a)** Volcano plots depicting shared gene expression profiles of bronchial epithelial cell cultures infected with SARS‐CoV, SARS‐CoV‐2 or MERS‐CoV for **(a)** 24, **(b)** 48 hpi or **(c)** 72 hpi in comparison to HCoV‐229E and HCoV‐OC43; *n* = 4. Red dots indicate significantly upregulated genes, and blue dots indicate significantly downregulated genes. **(d)** Heatmap of the overlap of significantly changed genes during 24, 48 and 72 hpi infection with SARS‐CoV‐2, SARS‐CoV, MERS‐CoV or HCoV‐229E or HCoV‐OC43 and mock as uninfected control. **(e)** Gene set variation analysis (GSVA) was performed on interferon response genes after 24, 48 and 72 hpi (*n* = 4) in highly pathogenic (orange) and low pathogenic (blue) coronavirus‐infected cultures. As HCoV‐OC43 infection was conducted at 33°C, SARS‐CoV‐2 infection at 33°C was included as control (pink). **(f)** IFN‐λ1 and **(g)** IFN‐α1 gene expression after 24, 48 and 72 hpi (*n* = 4) in highly pathogenic (orange) and low pathogenic (blue) coronavirus‐infected cultures. The error bar indicates the standard deviation. Significant differences in increased gene expression over time of infection (*P* < 0.05) are shown by the * symbol. Two‐way ANOVA followed by an unprotected Fisher's least significance difference test was conducted to test for significance. **(h)** ALI‐PBEC cultures were pretreated with 5 ng mL^−1^ interferon lambda (IFN‐λ1) for 60 min and subsequently infected with SARS‐CoV‐2 for 3 days with IFN‐λ1 present in the basal medium. Extracellular viral RNA copies are depicted in log scale. Data in **h** were analysed with a paired *t*‐test and are depicted as mean ± SEM. *n* = 4 independent donors.

To improve our understanding of why some coronaviruses are highly pathogenic while others are low pathogenic, we next compared transcriptional responses of epithelial cultures infected with highly pathogenic coronavirus strains (i.e. SARS‐CoV; SARS‐CoV‐2 and MERS‐CoV) with those infected with low pathogenic HCoV‐229E and HCoV‐OC43. We originally only included HCoV‐229E in the comparison, since HCoV‐OC43 does not infect bronchial epithelial cells at 37°C. However, to increase the power of our observations with low pathogenic coronaviruses, we next performed independent additional experiments at 33°C using the same donors but infected them with HCoV‐OC43 (see the Methods section). At 24 hpi, we detected five significantly changed genes with four genes decreased in expression and one gene increased. The four decreased genes included (AL022323.4 (KIAA1671)), a protein with—to our knowledge—no known function in the context of highly pathogenic coronaviruses and *CARNS1*, *EPX* and *MIR210HG*. The one gene that was increased in expression was *CLDN8*. At 48 and 72 hpi, we identified 397 and 744 genes significantly different, respectively (Figure 7a–d, Supplementary table 5). We observed that both HCoV‐229E and HCoV‐OC43 induced a stronger interferon response than coronaviruses that are highly pathogenic (Figure 7e). Notably, the experiments in which infection with HCoV‐OC43 was performed at 33°C also included SARS‐CoV‐2 as a control, to account for the possibility that the temperature affects the host immune response. When we investigated the expression of each interferon individually, IFN‐β1 was undetectable and no significant differences were observed in IFN‐α1; however, IFN‐λ1 was found to be expressed higher in HCoV‐229E and HCoV‐OC43 at 72 h than coronaviruses that are highly pathogenic (Figure 7f and g). This evasion of IFN responses has been described for highly pathogenic coronaviruses^17^, ^18^ and may explain why the host is more readily able to fight off an infection with HCoV‐229E or HCoV‐OC43. To support that this difference in gene expression profiles related to interferon has functional consequences for infection, we infected ALI‐PBEC with SARS‐CoV‐2 and treated these cultures with IFN‐λ1 right at the start of infection. We found that this indeed lowered the level of SARS‐CoV‐2 in the cultures as the IFN‐λ1 treatment led to > 1 log reduction in viral load in all four tested donors compared to the untreated infected cultures (Figure 7h). Together these data demonstrate that the suppression of IFNs by SARS‐CoV‐2 favors its replication, which is also likely for the other high pathogenic viruses.

## Discussion

Here, we report marked differences in the transcriptional response of primary human bronchial epithelial cells to infection with SARS‐CoV‐2 when compared to infection with other highly pathogenic and low pathogenic coronaviruses. Infection with SARS‐CoV, MERS‐CoV and low pathogenic HCoV‐229E evoked a partially overlapping transcriptional response, characterised by a significant and sustained increase in expression of IEGs such as *FOS*, *NR4A1* and *FOSB*. In contrast, the response to SARS‐CoV‐2 infection was characterised by the absence of such an increase, and the only DEGs in the SARS‐CoV‐2 dataset that were significantly downregulated at 72 hpi were *FOS*, *NR4A1* and *FOSB*. Infection with SARS‐CoV‐2 at a higher MOI and prolonged incubation, that is, for 7 days, did also not lead to an increase in the expression of the genes that were upregulated during SARS‐CoV, MERS‐CoV or HCoV‐229E infection. We furthermore confirmed the increased expression of these IEGs by MERS‐CoV and SARS‐CoV and the lack thereof by SARS‐CoV‐2 infection in publicly available datasets, including a clinical dataset. Treatment of infected cells with an NR4A1 antagonist decreased viral load, suggesting that the differential IEG gene expression may contribute to the kinetics of infection and potentially the pathogenicity of coronaviruses. Furthermore, we observed that activation of genes involved in interferon signalling was readily induced upon infection with the low pathogenic HCoV‐229E and HCoV‐OC43, whereas no such induction was observed with the three highly pathogenic coronaviruses, including SARS‐CoV‐2.

Where others have focused on the pathways that are activated by SARS‐CoV‐2 infection,^19^ the comparison between SARS‐CoV‐2 and other coronaviruses performed here highlights that SARS‐CoV‐2 may suppress or not induce expression in pathways that are activated by other coronaviruses. Many of the genes that were upregulated in cells infected with SARS‐CoV, MERS‐CoV and HCoV‐229E, but not SARS‐CoV‐2, were IEGs. There still remains a question as to whether SARS‐CoV‐2 actively suppresses expression of these genes or lacks the ability to induce their expression. IEGs are a group of genes with various functional activities that are coordinately and rapidly upregulated in response to a diverse set of stress or proliferation‐inducing stimuli. Their expression does not require *de novo* synthesis of proteins encoded by other genes, explaining their rapid induction. Besides being an IEG itself, recently it was established that *NR4A1* also regulates expression of IEGs^20^; in particular, NR4A1 suppresses expression of IEGs including *FOS* and *FOSB*. This demonstrates the complexity of the signalling regarding these IEGs. In our study, we treated Calu‐3 cells with the NR4A1 inhibitor DIM‐C‐pPhOH, thereby mimicking the putative SARS‐CoV‐2‐mediated suppression of NR4A1 activation and found reduced replication of both SARS‐CoV‐2 and MERS‐CoV. Nevertheless, it should be noted that despite the differences in IEG expression levels, the extent of infection was similar in all coronavirus‐infected cultures. Therefore, it is more likely that the suppression of IEGs is affecting the inflammatory/immune response following epithelial infection rather than the viral levels. Furthermore, several of these IEGs such as *FOS* and *FOSB* are related to the JNK/AP‐1 pathway, which has an essential role in cell death of infected cells via apoptosis and necrosis, and is important for the cellular response to pro‐inflammatory stimuli, and thus could limit progression of infection.^21^ It is additionally involved in innate and adaptive immunity through, for example, its relation to NF‐κB.^22^ The lack of the pro‐apoptotic function and regulatory role of the inflammation response of JNK/AP‐1‐related pathways could lead to prolongation of SARS‐CoV‐2 infection and/or delayed symptom development, especially early in infection. It may also contribute to the dysregulation of cytokine and chemokine responses. How SARS‐CoV‐2 exactly inhibits expression of IEGs will be of interest for further studies as the complexity of regulation of these IEGs requires in‐depth analysis. Activation of the JNK/AP‐1 signalling pathway also occurs in response to other respiratory viruses like influenza or RSV,^23^, ^24^ and small‐molecule inhibitors of this pathway were even shown to act as antivirals against influenza infection.^25^ Also, treatment with the NR4A1 agonist cytosporone B controlled Influenza A replication via regulation of antiviral responses in mice.^26^ Studying the role of IEGs in virus replication and infection biology is therefore interesting beyond coronaviruses.

Further research is needed to evaluate donor differences in the observed changes in gene expression in response to the variable coronaviruses. Our dataset only included a limited number of donors, with variable cellular composition. Unfortunately, we had to eliminate one donor from our dataset as it did not display transcriptional responses to infection with any of the viruses. We have repeated the SARS‐CoV‐2 infection with cells from the same donor, and then comparable responses were found between donors using targeted PCR reactions, suggesting that a technical issue in the RNA sequencing explained these findings with this donor. To overcome the limitation of having included only four donors, we validated the outcomes in publicly available datasets by assessing expression of the whole IEG gene set and several individual DEGs in these datasets. These analyses confirmed our findings. Importantly, the single‐cell RNA sequencing dataset confirmed that the absence of IEG expression that we detected in our SARS‐CoV‐2‐infected cultures was also present in samples from infected humans. We furthermore confirmed the lack of IEGs expression by SARS‐CoV‐2 in cultures from the additional donors (not included in the bulk RNA sequencing dataset) that were tested in the prolonged infection experiment, in which also more virus was used to infect the cells. We can, however, not exclude that – in addition to the observations reported in this study – additional differences may have been missed as a result of the relatively low number of replicates. We can also formally not exclude that the presence of a tumor in the lung tissue has affected the surrounding tumor‐free tissue from which the bronchial epithelial cells were isolated; however, obtaining lung tissue from healthy donors is a challenge for obvious reasons. We have validated our findings in datasets of others and could confirm our findings, and therefore we think that the use of this type of tissue has not affected our conclusions. The fact that we used a SARS‐CoV‐2 isolate from the first wave of the pandemic, and not the less pathogenic Omicron variant of SARS‐CoV‐2, can be viewed as a limitation. However, we specifically aimed to understand how highly pathogenic SARS‐CoV‐2 variants that cause severe disease redirect epithelial responses, also in view of preparedness for future pandemics.

We also aimed for functional translation of the effects found on interferon genes. However, to translate what we observed especially regarding the effect of modulation of NR4A1 and the JNK/AP‐1 pathway on coronavirus replication and pathogenesis, studies will need to be performed on primary cell cultures of more donors. To this end, future experiments should also include a larger range of viral doses to study the impact of initial infection severity on our findings. Finally, we largely focused on gene expression and our findings would likely benefit from larger scale proteomics approaches to gain further insights into the exact modulation of the signalling pathways by SARS‐CoV‐2, for example.

Our results also demonstrated an important difference in epithelial transcriptional responses after infection with pathogenic and low pathogenic coronaviruses. Induction of interferon‐related genes was less strong by the pathogenic coronavirus strains than low pathogenic HCoV‐229E and HCoV‐OC43. While an efficient interferon and interferon‐stimulated genes response was reported for HCoV‐229E and HCoV‐OC43,^27^ a lower response has been reported previously for pathogenic coronaviruses^28^, ^29^, ^30^ and is also less strong than pathogenic influenza A H1N1.^31^ This effect perhaps points to mechanisms of immune evasion through multiple possible mechanisms.^32^ When our cultures were supplemented with IFN‐λ1 during infection, reduced SARS‐CoV‐2 levels were observed, supporting a role for suppression of interferon‐mediated antiviral defences in the pathogenicity of coronaviruses such as SARS‐CoV‐2. Recently, Banday *et al*.^33^ showed similar results for SARS‐CoV‐2 using IFN‐β and IFN‐λ in a colon epithelial cell line (Caco‐2), while Feld *et al*.^34^ showed in outpatient COVID‐19 patients that treatment with pegylated interferon lambda resulted in a more rapid clearance for SARS‐CoV‐2 than the placebo group. Finally, also in bronchial epithelial cell cultures, this effect was confirmed using IFN‐β1 and IFN‐λ2.^35^

If we take all our results together, an interesting difference can be observed between low pathogenic coronaviruses, highly pathogenic coronaviruses with high mortality rates such as MERS‐CoV and SARS‐CoV, and SARS‐CoV‐2 that is somewhere in between those two severity levels. Low pathogenic coronaviruses show a quick induction of both interferon‐related genes and IEGs. Highly pathogenic MERS‐CoV and SARS‐CoV that have an increased mortality compared to SARS‐CoV‐2^36^ show a lack of interferon gene induction but a strong induction of IEGs, whereas SARS‐CoV‐2 lacks induction of both interferon‐related genes and IEGs. These changes could explain how low pathogenic strains are quickly cleared by a strong antiviral response, which combined with rapid induction of IEGs likely results in local inflammation‐related symptoms. On the one hand, MERS‐CoV and SARS‐CoV impair/delay antiviral defences, thereby extending infection time, which combined with a quick induction of IEGs, likely results in strong inflammation and related symptoms correlated to the high level of infection. Uniquely, SARS‐CoV‐2 also impairs antiviral responses; however, combined with a lack of IEG induction, inflammation is likely only local until the viral titres become so high that the resulting tissue damage promotes inflammation that leads to typical COVID‐19 symptoms. This is clearly speculation but in line with the severity of the viruses and translation of these findings into those in human subjects would be an exciting next step. Additionally, since SARS‐CoV‐2 is associated with higher levels of asymptomatic infections,^36^ it would be interesting to investigate whether in these patients the interferon response may be present while the induction of IEGs is still absent.^37^

The findings from our study highlight differences in transcriptional responses of airway epithelial cells to SARS‐CoV‐2 infection compared to other coronaviruses. The lack of induction or even suppression of IEGs expression by SARS‐CoV‐2 was striking and uncharacteristic of previously known coronaviruses and may contribute to its pathogenicity in multiple ways. In addition, lack of transcriptional activation of the interferon pathway is a unique feature that may distinguish highly pathogenic from low pathogenic coronavirus infections. This knowledge can aid in the understanding of SARS‐CoV‐2 pathogenesis and support development of therapeutic (host‐directed) strategies against coronaviruses in general.

## Methods

### Cell culture

Primary human bronchial epithelial cells (PBEC) were isolated from tumor‐free resected bronchus rings obtained from lung cancer patients undergoing a resection surgery at the Leiden University Medical Center (LUMC, Leiden, the Netherlands). Patients from which this lung tissue was derived were enrolled in the biobank via a no‐objection system for coded anonymous further use of such tissue (www.coreon.org). Within this framework, individual written informed consent is not needed. Since 1 September 2022, patients are enrolled in the biobank using written informed consent in accordance with local regulations from the LUMC biobank with approval by the institutional medical ethical committee (B20.042/Ab/ab and B20.042/Kb/kb). Donor baseline characteristics are provided in Supplementary table 1.

PBEC were biobanked as described,^38^ and the procedure is described in short below.

### Isolation and storage of primary bronchial epithelial cells

In short, after 2 h of incubation of the bronchial ring in PBS supplemented with protease XIV, cells were scraped off and seeded in Keratinocyte Serum‐Free Medium (KSFM, Life‐Technologies Europe B.V., the Netherlands) containing 0.2 ng mL^−1^ Epidermal Growth Factor (EGF; Gibco, USA), 25 μg mL^−1^ Bovine Pituitary Extract (BPE; Life Technologies Europe B.V.), 1 μm isoproterenol (Sigma‐Aldrich, USA) and 100 μg mL^−1^ primocin (Invivogen, the Netherlands) in 6‐well plates (Corning Costar, USA) coated with 30 μg mL^−1^ PureCol (Advanced BioMatrix, USA), 5 μg mL^−1^ human fibronectin (Promocell, Germany) and 10 μg mL^−1^ bovine serum albumin (Fraction V; Thermo Fisher Scientific, USA). After cell cultures had reached 80–90% confluence, cells were harvested using soft trypsin (Life Technologies Europe B.V.) and soybean trypsin inhibitor (SBTI; Sigma‐Aldrich) and stored in liquid nitrogen until further use.

### Primary bronchial epithelial cell culture on inserts

Cryopreserved PBEC were thawed and expanded in a coated T75 flask (Greiner Bio‐One, the Netherlands) until 80–90% confluency was reached after which cells were seeded in a 12‐well cell culture insert (40 000 cells per insert; Transwell®, Corning Costar), coated as described above. Apical and basal sides of inserts were filled with a B/D medium supplemented with 1 nm EC23 (Tocris, UK). B/D medium is a mix of 50% Bronchial Epithelial Cell Medium‐basal (BEpiCM‐b; ScienCell, Sanbio) and 50% Dulbecco's modified Eagle's medium (DMEM; Stemcell Technologies, Germany) supplemented with 12.5 mm HEPES, bronchial epithelial cell growth supplements, 100 U mL^−1^ penicillin, 100 μg mL^−1^ streptomycin (all from ScienCell) and 1 mm glutaMAX (Thermo Fisher Scientific). After confluency was reached, the apical medium was removed and cells were cultured at the air–liquid interface (ALI) in B/D medium with 50 nm EC‐23 for up to 5–6 weeks; medium was refreshed three times a week, each time the apical side was washed with pre‐warmed PBS.

### Cell culture of Calu‐3 cells

Calu‐3 cells (ATCC, HTB‐55TM) were cultured in Eagle's minimum essential medium (EMEM, Lonza), supplemented with 9% foetal calf serum (FCS; Capricorn Scientific, USA), 1% non‐essential amino acids (NEAA; Sigma‐Aldrich), 2 mm l‐glutamine (Sigma‐Aldrich), 1 mm sodium pyruvate (Sigma‐Aldrich) and 100 U mL^−1^ of penicillin/streptomycin (P/S; Sigma‐Aldrich). Infections were performed with the same medium, except that 2% FCS was used.

### Cell culture of Vero E6 cells

Vero E6 cells (Collection Medical Microbiology, LUMC) were cultured in DMEM with 4.5 g L^−1^ glucose with l‐glutamine (DMEM; Lonza, Switzerland), supplemented with 8% FCS and 100 U mL^−1^ of P/S. Infections in Vero E6 cells were performed in EMEM with 25 mm HEPES supplemented with 2% FCS (Capricorn Scientific, Germany), 2 mm l‐glutamine, and 100 U mL^−1^ of P/S.

### Cell culture of HUH‐7 cells

HUH‐7 cells (Collection Medical Microbiology, LUMC) were cultured in DMEM with 4.5 g L^−1^ glucose with l‐glutamine (DMEM; Lonza, Switzerland), supplemented with 8% FCS, 1% NEAA (Sigma‐Aldrich), 2 mm l‐glutamine and 100 U mL^−1^ of P/S. Infections in HUH‐7 cells were performed in EMEM with 25 mm HEPES supplemented with 2% FCS, 2 mm l‐glutamine and 100 U mL^−1^ of P/S. All cell cultures were maintained at 37°C in an atmosphere of 5% CO_2_.

### Virus stocks

All experiments with SARS‐CoV, SARS‐CoV‐2 or MERS‐CoV were performed at the LUMC biosafety level 3 facilities. The clinical isolate SARS‐CoV‐2/Leiden‐0008 was isolated from a nasopharyngeal sample collected at the LUMC during the first wave of the coronavirus pandemic in March 2020 (GenBank: MT705206.1). SARS‐CoV‐2/Leiden‐0008 (Passage 2) and SARS‐CoV isolate Frankfurt 1^39^ (Passage 4) were grown in Vero E6 cells. The SARS‐CoV‐2 stock was sequenced to exclude Vero cell adaptation in the spike S1/S2 cleavage site. MERS‐CoV (N3/Jordan, GenBank: KJ614529.1) (Passage 3) and HCoV‐229E (Passage 2, GenBank: NC_002645.1) were grown on HUH‐7 cells. HCoV‐OC43 was isolated from a bronchioalveolar lavage sample collected between 2018 and 2020 at the LUMC, and grown on primary bronchial epithelial cell cultures at the air–liquid interface. Next‐generation sequencing was performed, and the isolate was mapped to the HCoV‐OC43 strain ATCC VR‐759 (GenBank: AY585228.1). Virus titres were determined by plaque assay on Vero E6 cells, and for MERS‐CoV and HCoV‐229E on HUH7 cells, as described before.^40^ Virus titre of HCoV‐OC43 was determined by RT‐qPCR, due to the lack of susceptible cells for plaque assay.

### Viral infection of ALI‐PBEC

The apical side of inserts was washed with 200 μL PBS for 10 min at 37°C to remove excess mucus and basal medium was refreshed prior to infection. Cells were infected with 30 000 plaque‐forming units (PFU) of SARS‐CoV‐2, MERS‐CoV, SARS‐CoV and HCoV‐229E in 200 μL PBS per insert for 2 h at 37°C on a rocking platform (estimated multiplicity of infection [MOI] of 0.03).^41^, ^42^ For HCoV‐OC43, the experiments were performed in an independent experiment, however, using cultures from the same donors. Infections with HCoV‐OC43 were performed at 33°C and SARS‐CoV‐2 was taken along as a control at this temperature. The same multiplicity of infection for HCoV‐OC43 was used as for the other coronavirus infections, but the titre was based on RT‐qPCR measurement of the virus stock, in lack of a good cell culture model to perform a plaque assay experiment. For non‐infected (mock) controls, the same procedure was performed with PBS only. After removal of the inoculum, the apical side was washed three times with PBS, and cells were incubated at 33°C or 37°C until sampling at several time points post infection (p.i.). At each sampling time point, 200 μL of PBS was added to the apical side of the cells, and after incubation for 10 min at 37°C supernatant was harvested for quantification of infectious viral particles by plaque assay and RNA copies by RT‐qPCR. Cells were then lysed using 200 μL of RNA lysis buffer (Promega, the Netherlands) or 250 μL Guanidine thiocyanate (GITC) reagent (3 m GITC (Fluka), 2% sarkosyl (Sigma‐Aldrich), 50 mm Tris (Affymetrix) and 20 mm EDTA (Sigma‐Aldrich)) per insert. For long‐term SARS‐CoV‐2 infection, cells were infected with 10^6^ PFU of SARS‐CoV‐2 (estimated MOI of 1) in the same way as described above, but the inserts were incubated for 1–7 days.

### Viral load reduction assay

Compounds were purchased from MedChemExpress, USA (DIM‐C‐pPhOH, Cat. No. HY‐112055; Sp600125, Cat. No. HY‐12041; T‐5224, Cat. No. HY‐12270; Cytosporone B, Cat. No. HY‐N2148) and dissolved in DMSO. Calu‐3 cells were seeded in 24 wells at a density of 1.6 × 10^5^ cells per well in 500 μL of culture medium. After 24 h of culture, Calu‐3 cells were pretreated for 1 h with the compounds in infection medium and subsequently infected with 1.6 × 10^5^ PFU of SARS‐CoV‐2 or MERS‐CoV (MOI of 1) in 150 μL of infection medium with compounds. After 1 h, cells were washed three times with medium and 500 μL of medium with compounds was added. At 24 h post infection (hpi), supernatant was harvested to determine the viral load by quantifying the levels of extracellular viral RNA by RT‐qPCR. Intracellular RNA was collected by lysing the cells in 250 μL of GITC reagent. The potential cytotoxicity of the compounds was tested in parallel on uninfected cells in 96‐well plates using the CellTiter 96 Aqueous One Solution Cell Proliferation Assay (Promega). ALI‐PBEC were pretreated with 5 ng mL^−1^ recombinant human interferon λ1 (IFN‐λ1; R&D Systems, Minneapolis, USA) for 60 min, and further infected with SARS‐CoV‐2 (estimated MOI of 1) with the presence of IFN‐λ1 in the basal medium. RT‐qPCR was used to quantify extracellular viral RNA copies in the supernatant at 72 hpi.

### RNA isolation and quantitative real‐time PCR

Total intracellular RNA was robotically extracted using the Maxwell® 16 simply RNA tissue kit (Promega) and quantified using a NanoDrop™ One UV–Vis Spectrophotometer (Thermo Fisher Scientific) and stored at −80°C until further use. Extracellular RNA was extracted by magnetic bead isolation. Briefly, 20 μL of SpeedBeads™ carboxylate‐modified magnetic particles (Merck) and 135 μL of isopropanol were added to 100 μL of supernatant in a 96‐well plate. The plate was placed on a magnetic rack for 15 min, then supernatant was removed, and beads were washed one time with 150 μL of isopropanol and then two times with 200 μL of 70% ethanol. The beads were air‐dried and after removal of the plate from the magnetic rack, they were resuspended in 50 μL RNAse‐free water for 3 min. The plate was placed back on the magnetic rack for 10 min to collect the eluate containing total RNA.

For viral RNA measurement, the cellular reference gene *PGK1* served as control for intracellular RNA. Primers and TaqMan probes for *PGK1* were obtained from a published source.^40^ Viral RNA was quantified by RT‐qPCR using the TaqMan™ Fast Virus 1‐Step Master Mix (Thermo Fisher Scientific), with primer concentrations for SARS‐CoV‐2 and SARS‐CoV as described previously,^43^ for MERS‐CoV and HCoV‐229E with final primer concentrations of 450 nm each and probe concentration of 200 nm, and for HCoV‐OC43 with final primer concentrations of 1000 nm each and probe concentration of 166 nm. A standard curve of 10‐fold serial dilutions of a T7 RNA polymerase‐generated *in vitro* transcript containing the RT‐qPCR target sequences was used for absolute quantification. A RT‐qPCR programme of 5 min at 50°C and 20 s at 95°C, followed by 45 cycles of 5 s at 95°C and 30 s at 60°C, was performed on a CFX384 Touch™ Real‐Time PCR Detection System (Bio‐Rad, the Netherlands). For validation of other gene expression analyses, RNA was reverse‐transcribed and cDNA was amplified by real‐time qPCR (Bio‐Rad) using specific primers. Relative normalised gene expression compared to reference genes ATP synthase, H+ transporting, mitochondrial F1 complex, beta polypeptide (ATP5B) and ornithine decarboxylase antizyme 1 (OAZ1) were calculated according to the standard curve method. Reference genes were selected out of eight candidate reference genes using the ‘Genorm’ software (Genorm; Primer Design, Southampton, UK). Primer sequences are summarised in Supplementary table 2.

### RNA sequencing

The quality of total RNA was determined by bioanalyser and sequenced by GenomeScan (Leiden, the Netherlands) using an Illumina NovaSeq6000 sequencer with 20 million paired‐end reads per sample. The sequences were then trimmed using the Trimmomatic tool, version 0.33. Two consecutive computational strategies were applied for transcriptome reconstruction. First, the Spliced Transcripts Alignment to a Reference (STAR) version 2.5.3a was used to align and identify all reads that belong to the human genome (GRCh38). Samples were then mapped against respective virus genomes SARS‐CoV‐2 (NC_045512.2), MERS‐CoV (NC_019843.3), SARS‐CoV (AY291315.1), HCoV‐229E (NC_002645.1) and HCoV‐OC43 (NC_006213.1). Reads mapping to each gene for each virus were added together and normalised to the total number of reads including human‐mapped reads within a given sample using VST function in Deseq2 R package.

Originally cultures derived from five donors were sequenced; however, one donor did not show transcriptional responses to any of the viruses and was therefore excluded from the dataset.

### Differential expression analysis

RNA‐seq analysis was conducted using the R package EdgeR. Differential expression analysis was conducted comparing virus infection at each time point to time‐matched no virus control/mock using the following model (gene expression ~ patient ID + cell treatment). For high pathogenic virus analysis, SARS‐CoV, MERS‐CoV and SARS‐CoV‐2 were merged together as one group and compared to HCoV‐229E. Gene signatures were made using the Gene Set Variation Analysis (GSVA) package (version 1.44.5). Genes that were significantly increased in SARS‐CoV, MERS‐CoV and HCoV‐229E‐infected cultures across all time points FOS; NR4A1; FOSB; AC025259.3; EGR3; CYR61; EGR1; AC008894.3; RASD1; EGR2; ZFP36; CTGF; IL6 were used to make a general coronavirus signature. Additionally, a general IFN response signature (IFIT1; ISG15; IFI6; OAS1; OASL; IFI44; HERC5; MX2; HERC6; OAS3; DDX58; IRF7; SAMD9; HELZ2; USP18; DDX60; LAMP3; EPSTI1; PLSCR1; EIF2AK2; PARP12; PARP9; PARP14; BST2; TRIM22; IFITM3; IRF9) was used to investigate the overall IFN response of each coronavirus. We compared transcriptional responses of epithelial cultures infected with highly pathogenic coronavirus strains (i.e. SARS‐CoV, SARS‐CoV‐2 and MERS‐CoV) with those infected with low pathogenic HCoV‐229E and HCoV‐OC43 at 24, 48 and 72 h. Infection with HCoV‐OC43 was conducted at 33°C; hence, for signature analysis, SARS‐CoV‐2 infection at 33°C was used as a comparison. For all analyses, expression of a gene was considered significantly different with a Benjamini–Hochberg corrected *P*‐value < 0.05 and a Fold change > |1.5|. For the heatmaps, data were normalised using the function vst DeSeq2 package R.

### Cellular deconvolution

Cellular deconvolution of bulk RNA‐seq data was performed to estimate the proportions of different cell types (multi‐ciliated, secretory, rare cell types and basal cells) from the gene expression for bulk RNA‐seq dataset. This analysis was performed as previously described 16 and is summarised in the [Link], [Link] files. The resulting deconvolution predicted cell ratios were compared within each treatment, using a paired *t‐*test. Briefly, AutoGeneS software was used on the Human Lung Cell Atlas v1.0 dataset^44^ to select and filter 400 genes from highly variable ones. The selection was based on minimised correlation and maximised distance between clusters in which genes with the most stable results across cohorts were selected and used to infer major cell type proportions. The RNA‐seq data were subsequently normalised to counts per million (CPM), and highly variable (HV) genes (*N* = 5000) were selected. Bulk deconvolution on all samples was then conducted using the CIBERSORT support vector regression (SVR) method.^45^

### Comparative analysis with online datasets

The selected studies included the following. (1) A study that includes a dataset of transcriptional bronchial epithelial responses to MERS‐CoV. In this study, primary airway epithelial cells were infected with an MOI of 5 and harvested at 0, 12, 24, 36 and 48 hpi (‘Primary human airway epithelial cell transcriptome response to wild type MERS‐CoV (icMERS)’; GEO accession ID: GSE81909).^46^ (2) A study that includes a dataset of 2B4 cells, a clonal derivative of Calu‐3 cells, which were infected with SARS‐CoV (MOI 0.1) and analysed at 12, 24 and 48 hpi (‘Dynamic Innate Immune Responses of Human Bronchial Epithelial Cells against SARS‐CoV and DOHV infection’; GEO accession ID: GSE17400).^47^ (3) A study that includes a dataset of primary bronchial epithelial cells that were infected with SARS‐CoV‐2 (MOI 0.25) and analysed at 48 hpi (‘Primary Human Airway Epithelial Cultures infected with SARS‐CoV‐2’; GEO accession ID: GSE153970).^48^ (4) A study using primary alveolar epithelial type‐2 organoid cultures that were infected with SARS‐CoV‐2 at 1 × 10^4^ TCID50 per well and harvested at 48 hpi (‘SARS‐CoV‐2 infection of primary human lung epithelium for COVID‐19 modeling and drug discovery’; GEO accession ID: GSE155518).^49^ (5) A study that uses primary human bronchial epithelial cells, which were infected with 1.45 × 10^4^ PFU of SARS‐CoV‐2 per ALI well (MOI 0.1) and analysed at 24, 48, 72 and 96 hpi (‘Characterisation of the SARS‐CoV‐2 Host Response in Primary Human Airway Epithelial Cells from Aged Individuals’; GEO accession ID: GSE175779).^50^ We performed Gene Set Variation Analysis (GSVA) of FOS, NR4A1, FOSB, AC025259.3, EGR3, CYR61, EGR1, AC008894.3, RASD1, EGR2, ZFP36, CTGF and IL6 on two publicly available single‐cell datasets. The first dataset is single‐cell RNA‐seq data obtained from air–liquid interface (ALI) cultured cells infected with (*n* = 3) and without SARS‐CoV‐2 (*n* = 3).^7^ The second dataset is single‐cell nucleo‐seq data from frozen lungs of healthy donors (*n* = 7) and donors deceased with COVID‐19 (*n* = 20).^51^

### Statistics

Statistical analysis was performed in GraphPad PRISM 9.0.1 (GraphPad Software Inc., CA). Performed statistical tests are indicated in the figure captions. Data are shown as mean ± SEM of cultures derived from several donors and differences were considered significant at *P* < 0.05.

## Conflict of interest

The authors declare no conflict of interest.

## Author contributions

**Ying Wang:** Conceptualization; data curation; formal analysis; investigation; methodology; project administration; writing – original draft; writing – review and editing. **Melissa Thaler:** Conceptualization; data curation; formal analysis; investigation; methodology; project administration; writing – original draft; writing – review and editing. **Clarisse Salgado‐Benvindo:** Formal analysis; investigation; methodology. **Nathan Ly:** Formal analysis; methodology. **Anouk A Leijs:** Investigation; methodology. **Dennis K Ninaber:** Formal analysis; investigation; methodology. **Philip M Hansbro:** Investigation; resources; supervision; writing – review and editing. **Fia Boedijono:** Formal analysis; investigation. **Martijn J van Hemert:** Conceptualization; funding acquisition; investigation; resources; supervision; writing – review and editing. **Pieter S Hiemstra:** Conceptualization; funding acquisition; investigation; resources; supervision; writing – review and editing. **Anne M van der Does:** Conceptualization; funding acquisition; investigation; project administration; resources; supervision; writing – original draft; writing – review and editing. **Alen Faiz:** Conceptualization; formal analysis; investigation; methodology; project administration; resources; supervision; writing – original draft; writing – review and editing.

## Supporting information


Supplementary figures 1–4

Supplementary tables 1–5

## Data Availability

The datasets generated and/or analysed during the current study will be made available upon publication via the European Genome‐phenome Archive (EGA) but are currently available from the corresponding author on reasonable request.
