# Supplementary material for: SARS‐CoV‐2‐infected human airway epithelial cell cultures uniquely lack interferon and immediate early gene responses caused by other coronaviruses
Source: Clin Transl Immunology. 2024 Apr 15;13(4):e1503. doi: 10.1002/cti2.1503 (PMC11017760; doi:10.1002/cti2.1503)
Supplement: Supplementary file 1 — Supplementary figures 1–4 [file CTI2-13-e1503-s002.docx]

**Supplementary figures**

**SARS-CoV-2-infected human airway epithelial cell cultures uniquely lack interferon and immediate early gene responses caused by other coronaviruses**

Ying Wang^#1^, Melissa Thaler^#2^, Clarisse Salgado-Benvindo^2^, Nathan Ly^3^, Anouk A Leijs^2^, Dennis K Ninaber^1^, Philip Hansbro, Fia Boedijono, Martijn J van Hemert^2^, Pieter S Hiemstra^1^, Anne M van der Does^1^^, Alen Faiz^3^^*

**
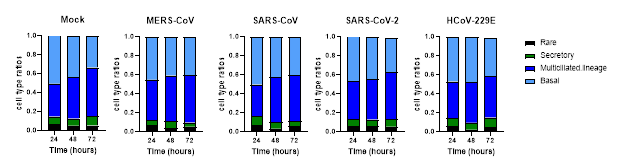
Supplementary figure 1. Cellular deconvolution during coronavirus infection of well-differentiated air-liquid interface (ALI) cultures of primary bronchial epithelial cells (PBEC).** PBEC that were differentiated for 6 weeks at ALI were infected in parallel with four different coronaviruses. Relative proportion of different cell types of infected cultures over 72h determined by cellular deconvolution of the transcriptomic datasets. N=4 independent donors.


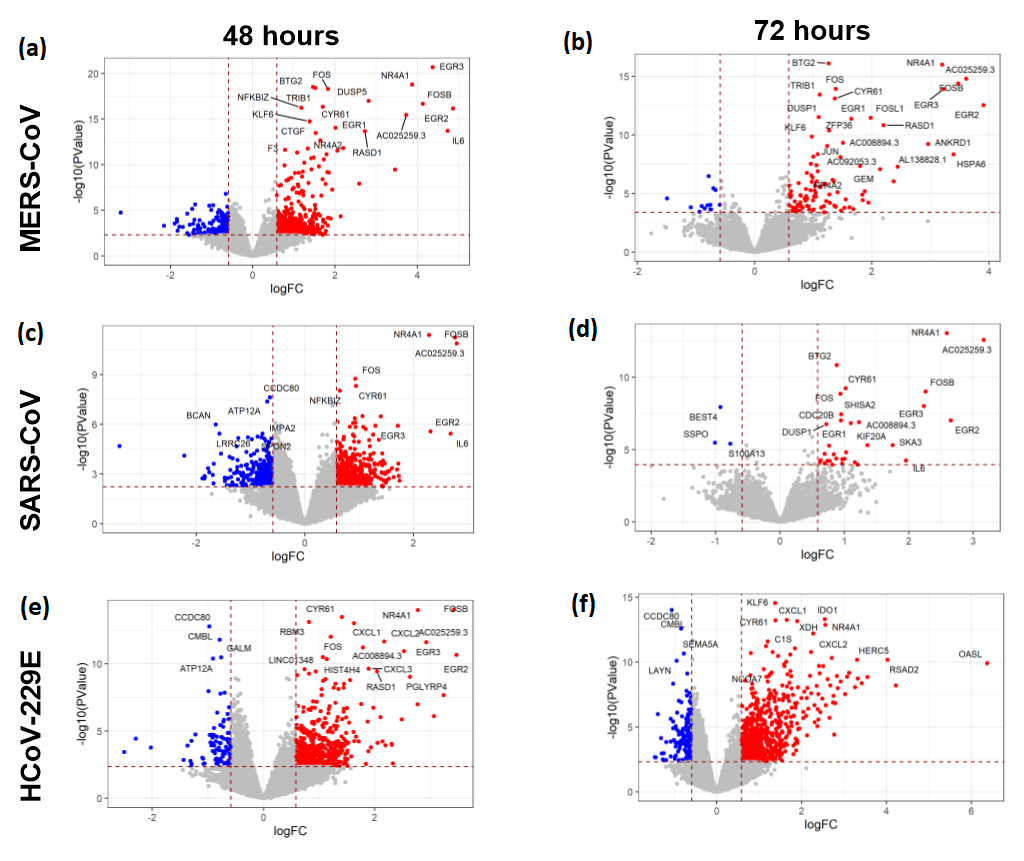


**Supplementary figure 2. Gene expression profiles of well-differentiated primary bronchial epithelial cell cultures infected with three different coronaviruses and analyzed at 48 and 72h post infection**

Volcano plots depicting gene expression profiles of bronchial epithelial cell cultures infected with (a)(b) MERS-CoV, (c)(d) SARS-CoV or (e)(f) HCoV-229E at 48 and 72 hpi, respectively in comparison to the uninfected controls. Red dots indicate significantly upregulated genes and blue dots indicate significantly downregulated genes. N=4 independent donors.


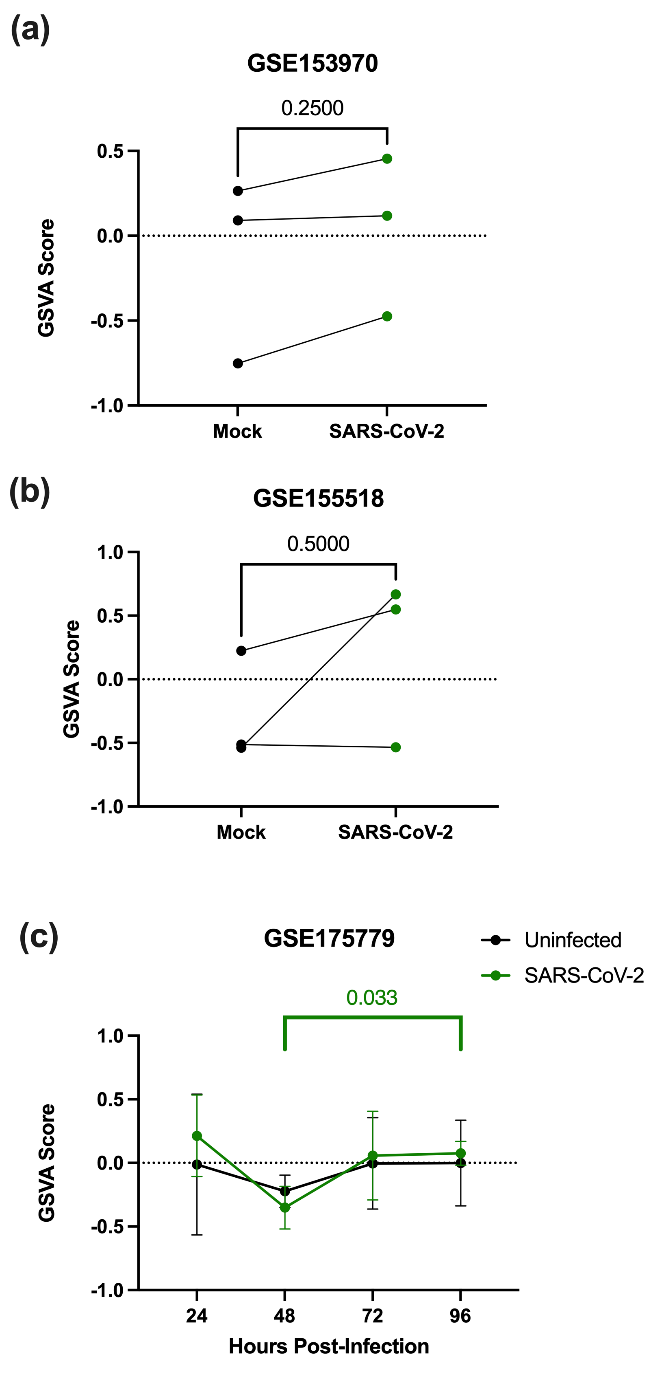
**Supplementary figure 3. GSVA of IEG gene set** on **(a)** A publicly available dataset (GSE153970) with RNA-seq analysis on primary epithelial cell cultures mock-infected or infected with SARS-CoV-2 at a MOI=0.25 at 48 hpi. **(b)** A publicly available dataset (GSE155518) derived from primary human lung alveolar epithelial organoid cultures infected with SARS-CoV-2 and assessed at 48 hpi. **(c)** A publicly available dataset (GSE175779) from primary human bronchial epithelial cells infected with SARS- CoV-2 over a 96 h infection period. Wilcoxon paired nonparametric statistical analysis was conducted on GSE153970 and GSE155518, while two-way ANOVA with Bonferroni correction was performed on GSE175779.

**
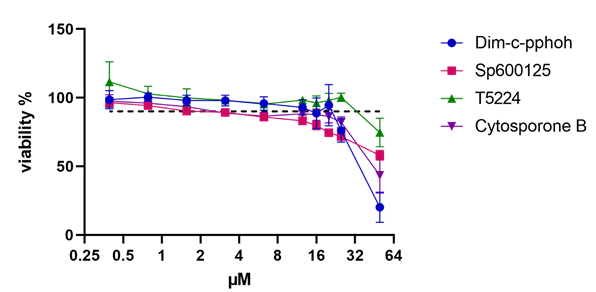
Supplementary figure 4. Cytotoxicity of tested JNK/AP-1 signaling modulating compounds on Calu-3 cells.**

Cell viability was measured by MTS assay after 24h of treatment. The black dotted line indicates 90% viability. Mean values ± SD are shown, n=4.
